# Burden and seasonality of medically attended influenza like illness (ILI) in Ethiopia, 2012 to 2017

**DOI:** 10.1186/s12879-020-4827-0

**Published:** 2020-02-18

**Authors:** Musse Tadesse, Mesfin Mengesha, Adamu Tayachew, Desalegn Belay, Abdulhafiz Hassen, Abyot Bekele Woyessa, Etsehiwot Zemelak, Berhan Beyene, Wubayehu Kassa, Workenesh Ayele, Bethel Teshome, Mikias Mekonen, Zewdu Assefa, Beyene Moges

**Affiliations:** Center for Public Health Emergency Management, Ethiopian Public Health Institute, PO BOX 1242, Addis Ababa, Ethiopia

**Keywords:** Ethiopia, Incidence rate, Influenza, ILI, SARI, Seasonality

## Abstract

**Background:**

The influenza virus spreads rapidly around the world in seasonal epidemics, resulting in significant morbidity and mortality. Influenza-related incidence data are limited in many countries in Africa despite established sentinel surveillance. This study aimed to address the information gap by estimating the burden and seasonality of medically attended influenza like illness in Ethiopia.

**Method:**

Influenza sentinel surveillance data collected from 3 influenza like illness (ILI) and 5 Severe Acute Respiratory Illness (SARI) sites from 2012 to 2017 was used for analysis. Descriptive statistics were applied for simple analysis. The proportion of medically attended influenza positive cases and incidence rate of ILI was determined using total admitted patients and catchment area population. Seasonality was estimated based on weekly trend of ILI and predicted threshold was done by applying the “Moving Epidemic Method (MEM)”.

**Result:**

A total of 5715 medically attended influenza suspected patients who fulfills ILI and SARI case definition (77% ILI and 23% SARI) was enrolled. Laboratory confirmed influenza virus (influenza positive case) among ILI and SARI suspected case was 25% (1130/4426) and 3% (36/1289). Of which, 65% were influenza type A. The predominantly circulating influenza subtype were seasonal influenza A(H3N2) (*n* = 455, 60%) and Influenza A(H1N1)pdm09 (*n* = 293, 38.81%). The estimated mean annual influenza positive case proportion and ILI incidence rate was 160.04 and 52.48 per 100,000 population. The Incidence rate of ILI was higher in the age group of 15–44 years of age [‘Incidence rate (R) = 254.6 per 100,000 population’, 95% CI; 173.65, 335.55] and 5–14 years of age [R = 49.5, CI 95%; 31.47, 130.43]. The seasonality of influenza has two peak seasons; in a period from October–December and from April–June.

**Conclusion:**

Significant morbidity of influenza like illness was observed with two peak seasons of the year and seasonal influenza A (H3N2) remains the predominantly circulating influenza subtype. Further study need to be considered to identify potential risks and improving the surveillance system to continue early detection and monitoring of circulating influenza virus in the country has paramount importance.

## Background

Influenza is an acute viral respiratory tract disease in humans, often characterized by fever, headache, myalgia, prostration, coryza, sore throat and cough [1]. The influenza virus spreads rapidly around the world in seasonal epidemics, resulting in significant morbidity and mortality [2–8]. Globally, an estimated 3 to 5 million cases of severe influenza illness and 291, 243–645, 832 seasonal influenza-associated respiratory deaths occur annually [9, 10]. A higher burden of influenza associated hospitalization has been reported among African children compared to children in other Regions [2–8].

Seasonal influenza typically occurs every year in the late fall or winter in temperate regions [11] while the seasonality is less clearly defined in tropical and subtropical regions [12]. Seasonality patterns of influenza in eastern Africa including Ethiopia, have not been clearly established [13]. However, some evidences from different studies indicated that Influenza A (H1N1) pdm2009, Seasonal Influenza A (H3N2) and Influenza B are circulating in different countries of sub-Saharan Africa [14–16].

Estimates of the national burden of influenza associated hospitalization across age groups are severely limited in Africa. It was described only in few countries for influenza and influenza-related incidence data remain inadequate [3, 17, 18]. World Health Organization (WHO) has highlighted the need for influenza disease burden estimates especially from low- and middle-income countries. These estimates would enable governments to make informed evidence-based decisions when allocating scarce resources and planning intervention strategies to limit the impact and spread of the disease [19]. This study aimed to address this gap by estimating the burden and seasonality of influenza like illness using sentinel surveillance data collected from 2012 to 2017 in Ethiopia.

## Method

### Data source

**Fig. 1:**
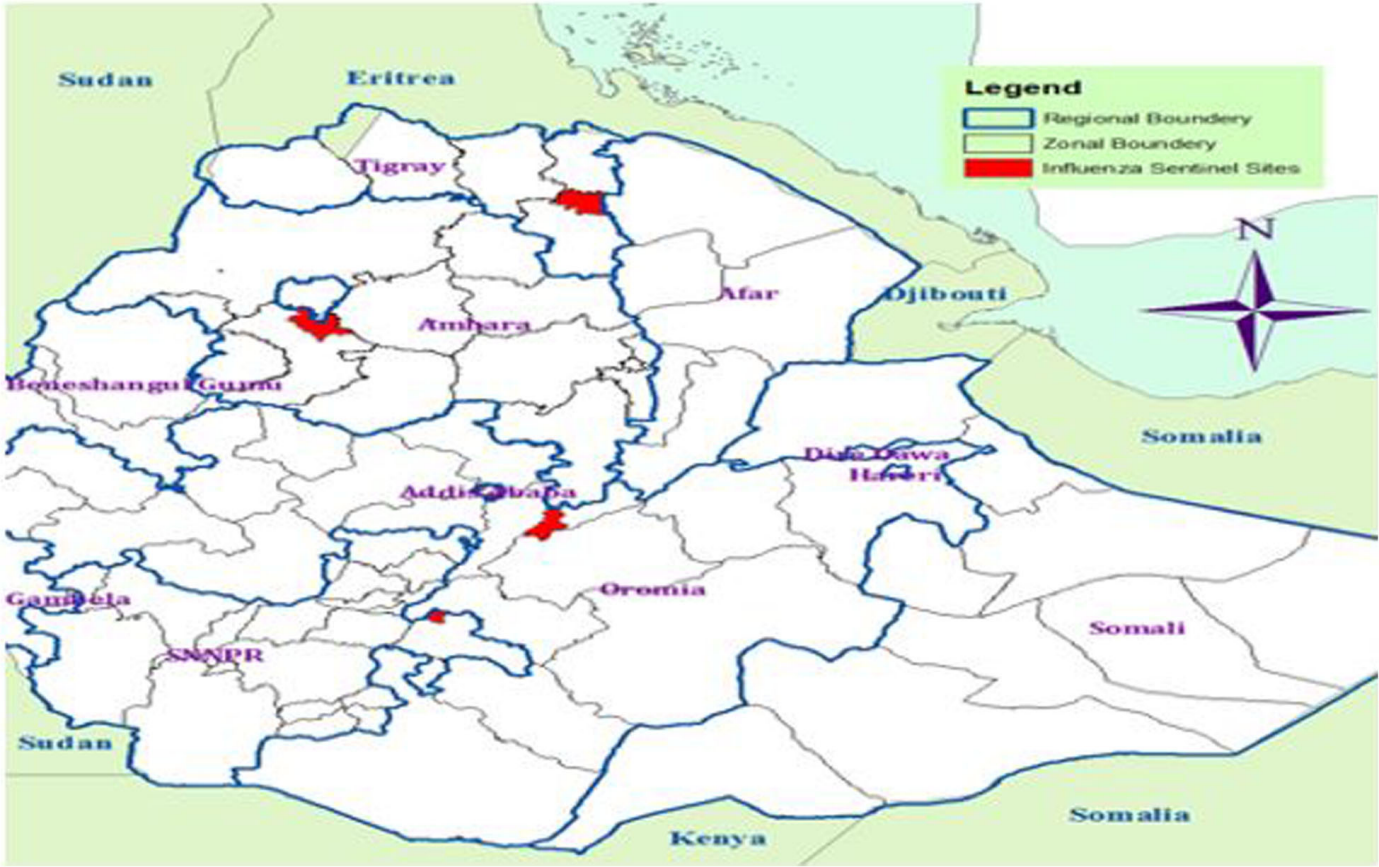
Geographical location of ILI and SARI sentinel surveillance sites in Ethiopia

The Influenza like Illness (ILI) and Sever Acute Respiratory Illness (SARI) surveillance data collected in a period from 2012 to 2017 was applied to estimate proportion of laboratory confirmed influenza positive ILI cases (medically-attended) among patients presented to the sentinel sites (health facility) seeking medical care for any kind of illness and influenza positive ILI cases among the catchment population (incidence rate) in Ethiopia. The ILI program is a health center-based sentinel surveillance system that monitors children and adults presenting with sudden onset of fever > 38 °C and cough or sore throat. The three ILI sentinel sites (Shiromeda health center which was established in 2008, Kolfe health center and Akaki health center established in 2010) are located in Addis Ababa. The SARI program is a hospital-based sentinel surveillance system that monitors children and adults hospitalized with pneumonia across five hospitals in Ethiopia. The SARI sentinel sites are located in four regions which are Yekatit-12 hospital (Addis Ababa) established in 2008, Mekele hospital (Tigray region, northern Ethiopia), Felege-hiwot hospital (Amahara region, North weastern Ethiopia), Adama hospital (Oromia region, Central Ethiopia) and Adare hospital (Southern region of Ethiopia) which was established in 2012; Fig. 1, below.

### Case definitions and data collection

ILI was defined as any person presenting with sudden onset of fever ≥38 °C (axillary measured) AND cough or sore throat in the absence of other diagnosis with in the last 7 days [17, 18]. SARI was defined as any person > 5 years old presenting with symptoms of acute lower respiratory infection with sudden onset of fever ≥38 °C, AND cough or sore throat, AND shortness of breath or difficulty breathing with in the last 7 days, AND requiring hospitalization [17, 18] or any child ≤5 years that fulfilled the case definition of pneumonia and severe pneumonia as per the Integrated Management of Childhood Illnesses protocol [19]. Therefore, the incidence rate of ILI was defined as the number of new ILI cases (fulfilling ILI case definition) per total population of the catchment area per year.

### Epidemiological and laboratory data

Epidemiological data and specimens were collected from patients who presented at a sentinel site and fulfill the case definition for ILI and SARI. Epidemiological data including patients’ demographic information was recorded on a standard case based reporting format and weekly aggregated data format. The case based reporting format has every detailed epidemiological and laboratory based information for those sampled cases. Whereas the aggregated data form was collected a weekly bases with a components of total admission, number of new case, number of sampled, number of tested, number of positive and proportion of both ILI and SARI case by specific age group weekly aggregate collected from each sentinel sites. Overall data of Influenza surveillance from 2012 to 2017 was obtained from Ethiopian Public Health Institute with an official request.

Each sentinel site has a protocol to collect throat swab specimens as part of sentinel surveillance system. The sentinel sites focal persons obtained unwritten (Verbal) consent from each patient prior to collection of throat swab specimen. Since it’s a routine surveillance activity under the health care delivery system only verbal consent from the patients are the only requirement. Throat swab samples were systematically collected from out-patients of all ages who fulfilled the case definition for ILI per week at sentinel surveillance sites. Similarly throat swab samples were also collected from patients who fulfilled the SARI case definition and admitted to designated SARI sentinel surveillance sites (hospital). Specimen were collected within 7 days after the first onset of symptoms for ILI and SARI. Throat swabs samples was placed in viral transport media (VTM) and stored at 4 °C until transported to the National Influenza Laboratory (NIL) at Ethiopian Public Health Institute (EPHI). Shipment of throat swab specimens in viral transport media to the NIL at EPHI was conducted within 72 h of collection using a cold chain system. Viral RNA from throat swabs were extracted and subjected to real-time PCR amplification with parameters set for influenza testing, according to Center for disease Control and Prevention (CDC) protocol using reagents obtained from Influenza Reagent Resources (IRR). Further sub-typing and characterization of influenza A-positive specimens were carried out using the CDC real-time reverse transcription PCR protocol [20].

### Population denominators

The total admitted patient in each sentinel sites for health care service and the population in the lower administrative level where the sentinel sites are located (district/woreda population) was applied as a denominator to calculate the medically attended ILI positive case proportion and incidence rate. The total admitted population was obtained from the weekly aggregated data collected from the registration log book for all patients seeking any medical service in each health facilities and used as a denominator population to calculate the influenza positive case proportion of ILI and SARI associated influenza patients. Age- and year-specific total population denominators were obtained from projections of the 2007 census data for Ethiopia reported by Central Statistical Authority [CSA, 2007]. The midyear population size were applied for calculation of the Incidence All estimates were obtained overall and within the age categories of: < 1 years, 1–4 years, 5–14 years, 15–44 years and > 44 years of age. This age category has different from WHO age category of 0 to < 2 years, 2 to < 5 years, 5 to < 15 years, 15 to < 50 years, 50 to < 65. The variation in age category applied in this manuscript was done by considering the availability of population denominator data from central statistical authority.

### Statistical analysis

The overall data management and analysis were done using Excel v-13, SPSS V-23 and RStudio (version 3.4.3) applications. The case based surveillance data and weekly aggregated data (aggregated by age specific total admission, number of new cases, number of tested and sampled per week) from 2012 to 2017 were entered and cleaned using excel v-13. The incomplete data set (missing values) and the outbreak investigation results in 2016 were excluded and only the complete data sets recorded in the routine sentinel surveillance system were included to maintain the consistency of surveillance data.

Descriptive statistics were carried out using SPSS V-23, for the basic demographic characteristics for calculating the frequency, percentage, mean, median and range. Cross tabulation also applied to identify the association between the case classification (ILI and SARI) of influenza positive cases by the determinant factors like sex and age; and also for the influenza types and sub-types.

The estimated medically attended influenza positive ILI case proportion was calculated by taking the influenza (ILI and SARI) positive cases divided by the total admission (cumulative from eight sentinel sites) extracted from the weekly aggregated data. The estimated medically attended influenza like illness incidence rate was calculated by taking the influenza positive cases divided by the cumulative population of the lower administrative level population size where each sentinel sites were located and multiplied by a constant of 100,000. The Incidence rate was calculated for each year of the surveillance period and the cumulative Incidence rate was calculated by taking the cumulative influenza positive cases (2012–2017) divided by the mid-year population. Incidence rates were expressed per 100,000 population.

Weekly trend of seasonal influenza with in the 7 year period was applied for seasonality and predicted threshold was done by applying the “Moving Epidemic Method (MEM)” using R-Studio version 3.4.3. As initial step, the weekly trend of medically attended influenza positive cases were done using excel v-13. Then the weekly Incidence rate of medically attended influenza like illness was for each year in the surveillance period (2012–2017) was applied. Since Ethiopia is located in the Northern hemisphere (4 degree); the Incidence rate on week 40 was used as a starting period and week 20 as the end period. The seasonality and MEM model was extracted from the analysis result on the R-Studio. This includes the epidemic threshold, high and very high threshold, starting and ending season of the epidemics including the goodness and intensity parameters.

The main outcome of this manuscript includes estimation of the burden, seasonality and predicted threshold of medically attended influenza positive ILI case and can be described as follows:

1. Burden of influenza positive ILI cases (Medically attended) was expressed in terms of proportion and Incidence rate as recommended by WHO-guideline.
  Proportion of influenza positive ILI cases = (Laboratory confirmed influenza virus positive ILI cases divided by total number of patients presented to the sentinel sites (health facility) seeking medical care for any kind of illness within a specified period)*100
  Incidence of ILI = (Laboratory confirmed influenza virus positive ILI cases divided by total population in the catchment area)* 100,000.
2. Seasonality was described as the peak season (period) of the year where the number of laboratory confirmed influenza positive cases of ILI were relatively high from the weekly trend of medically attended ILI cases.
3. Predicted threshold: this can be described as the expected incidence rate of laboratory confirmed influenza positive cases of ILI with in the specified time of the year.
  Epidemic threshold: defined as the predicted incidence rate of laboratory confirmed influenza positive case beyond the expected level and considered as an epidemics.

**Fig. 2:**
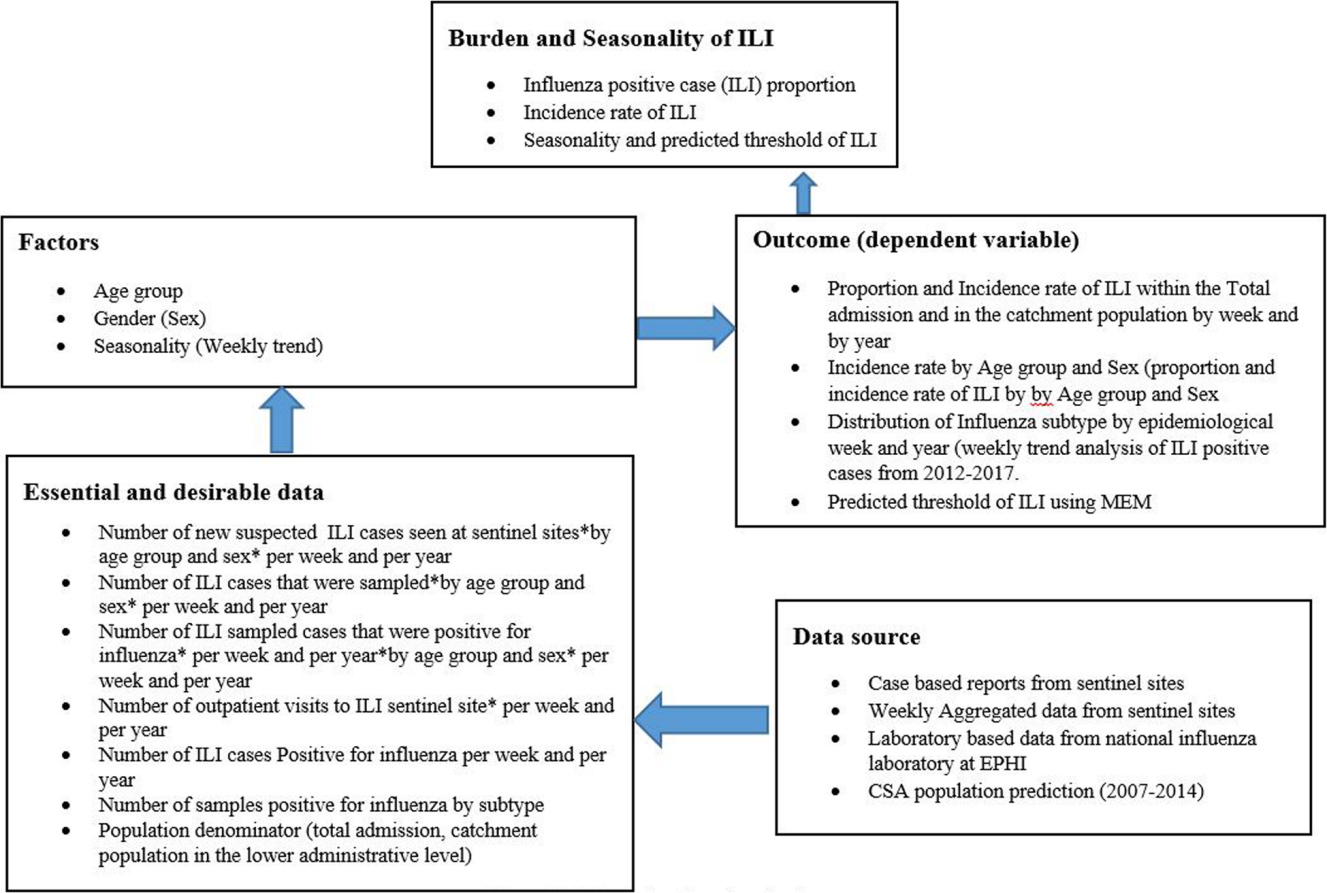
Schematic plan of analysis

This analysis was done using data collected as part of routine surveillance activities, and as such, ethical approval was deemed not necessary from the Ethiopian Public Health Institute’s Scientific and Ethical Review Office (SERO). The schematic analysis plan is presented in Fig. 2 below.

## Result

### Demographic characteristics and distribution of medically attended influenza (ILI and SARI) cases enrolled

A total of 5715 medically attended influenza suspected patients who fulfills ILI and SARI case definition was enrolled in eight influenza sentinel surveillance sites located in four regions of Ethiopia from 2012 to 2017. The majority of the cases enrolled in the sentinel surveillance system was outpatients with ILI (*N* = 4426, 77%) from 3 sentinel sites and the rest were inpatients presented with SARI (*N* = 1289, 23%) from 5 sentinel sites. Higher number of ILI cases was from Shiromeda Health Center (*n* = 2268, 51%) followed by Kolfe Health Center (*n* = 1843, 42%); all ILI sites are located in Addis Ababa. Forty percent (*n* = 2273) of the cases were under 15–44 years age group, followed by age group of 5–14 year (*n* = 1490, 26%) and higher number of cases were Females (*n* = 3274, 57%). Of the total 5715 medically attended influenza suspected cases 20% (*n* = 1166) was found positive for influenza and 65% (*n* = 755) of the positive cases were found Influenza A, Table 1 above.

**Table 1:** Characteristics of the patients enrolled at eight influenza surveillances sites in Ethiopia, 2012–2017

Variables	ILI Cases (*N* = 4426, 77.4%)		SARI Cases (*N* = 1289, 22.6%)		Total (*N* = 5715)	
	n	%	n	%	n	%
ILI Sentinel site (Health centers)						
Shiromeda Health Center	2268	51%	–	–	2268	40%
Kolfe Health Center	1843	42%	–	–	1843	32%
Akaki Health Center	315	7%	–	–	315	6%
SARI Sentinel Sites (Hospitals)						
Adama Teaching Hospital	–	–	50	4%	50	1%
Adare Hospital	–	–	147	11%	147	3%
Felege-Hiwot Hospital	–	–	291	23%	291	5%
Mekele Hospital	–	–	384	30%	384	7%
Yekatit 12 Hospital	–	–	417	32%	417	7%
Age group						
< 1 years	88	2%	482	38%	570	10%
1-4 years	412	9%	470	37%	882	15%
4-15 years	1296	29%	194	15%	1490	26%
15-44 years	2203	50%	70	6%	2273	40%
> 44 years	390	9%	32	3%	422	7%
Missing	55	1%	23	2%	78	1%
Sex						
Female	2695	61%	579	46%	3274	57%
Male	1749	39%	692	54%	2441	43%
Influenza positive						
Influenza A	724	64%	31	86%	755	65%
A(H3N2)	433	60%	22	71%	455	60%
A(H1N1)pdm09	284	39%	9	29%	293	39%
Not subtyped	7	1%	0	29%	7	1%
Influenza B	406	36%	5	14%	411	35%
Total influenza positive	1130	25%	36	3%	1166	20%

### Distribution of medically attended ILI and SARI cases by influenza type and sub-type

Of the total 1166 medically attended influenza positive cases (patients) 65% were influenza type A and 35% were influenza type B. Of the influenza type A cases the predominantly circulating influenza subtype (*n* = 455, 60%) were Seasonal Influenza A(H3N2) and (*n* = 293, 39%) were Influenza A(H1N1)pdm09. Among 4444 out-patient cases presented to three sentinel sites with Influenza-like-illness (ILI), 25% (*n* = 1130) were tested positive for influenza. Of which, the majority of patients who tested positive for influenza were influenza type A (*n* = 724, 64%), and of those, the majority were seasonal influenza A (H3N2) (*n* = 433, 60%). On the other hand, among 1271 (22% of all cases) in-patients presented to five SARI sentinel sites with Sever Acute Respiratory 36 (3%) were tested positive for influenza. The overall percent positive for medically attended influenza cases were 20% (1166/5715). Off the total influenza positive cases 27% (*n* = 399/1166) were within the age group of 5–14 years of age followed by 15–44 age groups (*n* = 573, 25%). One percent (*n* = 78) of influenza positive cases were missing patient age (Table 2, below).

**Table 2:** The number and proportion of medically attended influenza positive cases by age group, influenza case classification, and influenza subtype in Ethiopia, 2012–2017

Case Classification	Age group (y)	Number of specimens tested	Influenza Positive		Influenza A		Seasonal Influenza A(H3N2)		Influenza A(H1N1)pdm09		Influenza B	
			n	%	n	%	n	%	n	%	n	%
ILI	< 1	88	9	10%	5	56%	4	80%	1	20%	4	44%
	1–4	412	49	12%	36	73%	16	44%	18	50%	14	29%
	5–14	1296	395	30%	226	57%	135	60%	90	40%	168	43%
	15–44	2203	572	26%	391	68%	235	60%	155	40%	181	32%
	> 44	390	91	23%	58	64%	39	67%	18	31%	33	36%
	Missing	55	14	25%	8	57%	4	50%	2	25%	6	43%
	Total	4444	1130	25%	724	64%	433	60%	284	39%	406	36%
SARI	< 1	482	11	2%	10	91%	5	50%	5	50%	1	9%
	1–4	470	17	4%	15	88%	12	80%	3	20%	2	12%
	5–14	194	4	2%	4	100%	3	75%	1	25%	0	0%
	15–44	70	1	1%	1	100%	1	100%	0	0%	0	0%
	> 44	32	3	9%	1	33%	1	100%	0	0%	2	67%
	Missing	23	0	0%	0	0%	0	0%	0	0%	0	0%
	Total	1271	36	3%	31	86%	22	71%	9	29%	5	14%
Total	< 1	570	20	4%	15	75%	9	60%	6	40%	5	25%
	1–4	882	66	7%	51	77%	28	55%	21	41%	16	24%
	5–14	1490	399	27%	230	58%	138	60%	91	40%	168	42%
	15–44	2273	573	25%	392	68%	236	60%	155	40%	181	32%
	> 44	422	94	22%	59	63%	40	68%	18	31%	35	37%
	Missing	78	14	18%	8	57%	4	50%	2	25%	6	43%
	Total	5715	1166	20%	755	65%	455	60%	293	39%	411	35%

### Medically attended influenza like illness (ILI) positive case proportion, 2012–2017

**Fig. 3:**
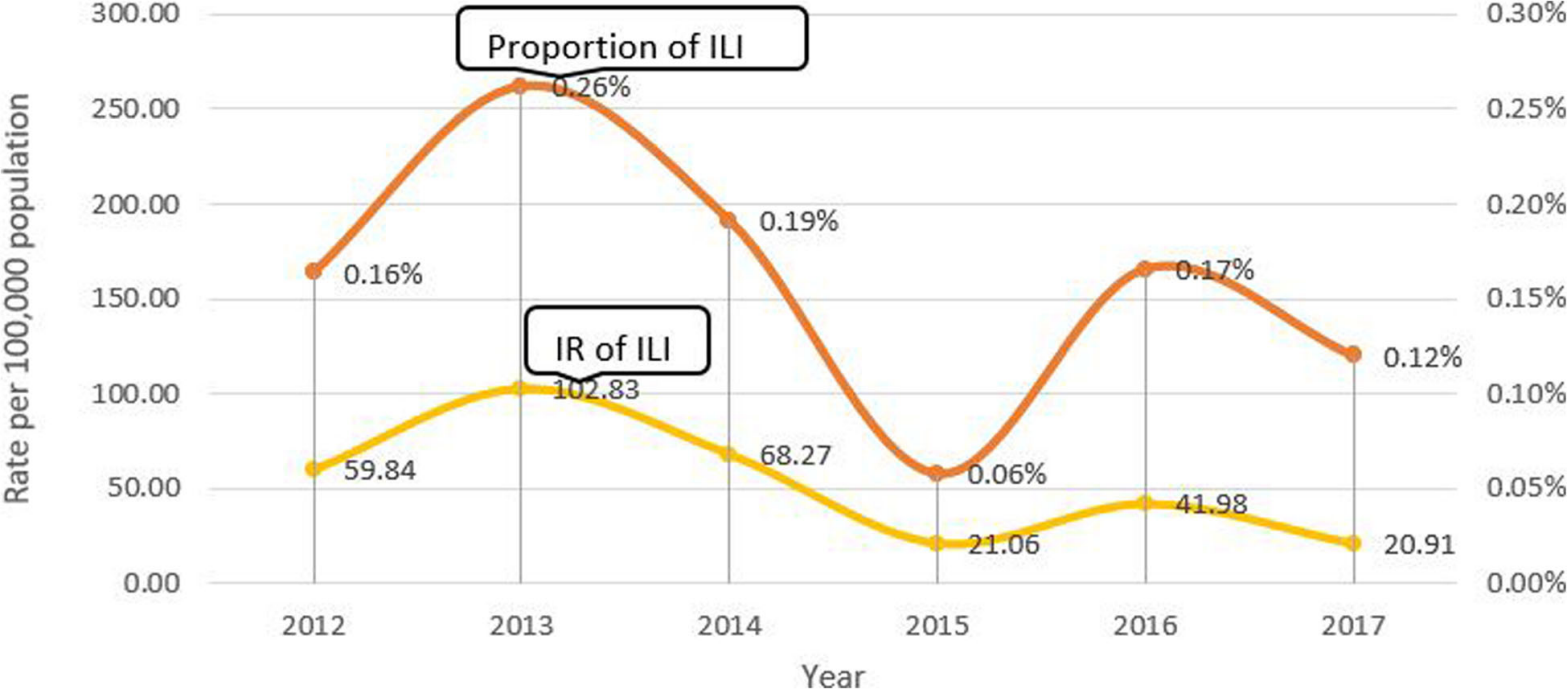
Medically attended influenza positive case (ILI) proportion and incidence rate per 100, 000 population from 2012 to 2017, Ethiopia

The overall proportion of laboratory confirmed influenza positive medically-attended ILI cases among patients presented to the sentinel sites (health facility) seeking medical care for any kind of illness (total admission rate) were 0.82% (824.6 per 100, 000 total admitted patients [95%CI; 44.39, 1604.79]).The mean annual proportion was 0.16% (160.04 per 100, 000 of total admitted patients). The higher influenza positive case proportion was recorded in 2013 by 0.26% (R = 261.7; 95%CI; 6.57, 516.76] followed by an influenza positive case proportion of 0.2% (R = 191.1 in 2014 [95% CI; 48.35, 333.95] per 100,000 population of total admission (Fig. 3, below). Medically attended influenza positive case proportion were high (2.5%) in the age group between 5 and 14 years of age [R = 2496.1, 95% CI; 1715.92, 3276.32] followed by 1% in the age group of 15–44 [R = 1009.0, 95% CI; 228.80, 1789.20] per 100,000 of total admitted patients (Table 3, below). Figure 3 below illustrates the proportion of medically attended ILI cases (number of influenza positive cases per total admitted patients) and incidence rate of medically attended ILI per 100, 000 population in the catchment area.

**Table 3:** Rate of laboratory confirmed influenza positive medically-attended ILI cases among patients presented to the sentinel sites (health facility) seeking medical care for any kind of illness (per 100,000 total admission rate), 2012–2017

Age group	2012		2013		2014		2015		2016		2017		2012–2017	
	Rate	95%CI	Rate	95%CI	Rate	95%CI	Rate	95%CI	Rate	95%CI	Rate	95%CI	Rate	95%CI
< 1 years	8	1.12, 56.7	54.9	26.2, 115.2	60.1	28.6, 126.1	14.6	3.6, 58.4	11.4	1.6, 80.9	36.9	9.2, 147.5	185.2	119.1, 287.1
1–4 years	86.5	52.1, 143.4	135.2	90.6, 201.7	71.2	39.4, 128.6	38.8	17.4, 86.4	64.8	32.4, 129.6	20.9	5.2, 83.6	450.6	354, 573.5
5–14 years	412	329.5, 515.1	759.2	646.9, 890.9	455.5	369.2, 562	132.8	88.2, 199.8	421.5	320.3, 554.6	123	68.1, 222.1	2496.1	2262.8, 2753.4
15–44 years	184	152.4, 222.2	246.5	211.6, 287.1	184.9	154.6, 221.1	64.8	48.2, 87.1	173.6	140.2, 215	153.2	116.7, 201	1009	929.7, 1095.0
> 45 years	31.8	15.6, 66.7	86.7	56.5, 133	120.9	84, 174	23.6	10.6, 52.5	77.9	47, 129.2	116.4	71.3, 190	73.1	59.7, 89.4
Total	163.8	143, 187.6	261.7	236.2, 289.9	191.1	169, 216.1	58	46.6, 72.1	165.6	141.8, 193.4	120.1	96.9, 148	824.6	778.3, 873.6

### Incidence rate of medically attended influenza like illness (ILI), 2012–2017

**Fig. 4:**
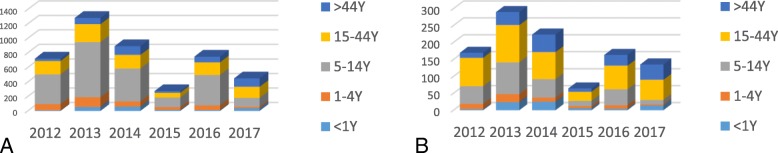
Medically attended influenza positive case among the total admission (**a**) and incidence rate (**b**) of ILI per 100, 000 population by age group, 2012–2017, Ethiopia

The overall incidence rate of medically attended laboratory confirmed influenza virus ILI cases in the lower administrative level catchment population from 2012 to 2017 were 324.1 per 100,000 population [CI 95%; 243.10, 405.00]; Fig. 4, below. The mean annual Incidence rate of ILI were 52.48 and higher in 2013 [R = 102.8, CI 95%; 70.72, 134.94] followed by Incidence rate of 68.3 [95% CI; 47.48, 89.06] by the year 2014 per 100, 000 population in the catchment area. The overall Incidence rate of ILI were high in the age group of 15–44 years of age [R = 254.6, 95% CI; 173.65, 335.55] followed by 5–14 years of age [R = 49.5, CI 95%; 31.47, 130.43] as indicated in Table 4 below.

**Table 4:** Incidence rate of medically attended Influenza like Illness cases per 100,000 population, 2012–2017

Age group	2012		2013		2014		2015		2016		2017			2012–2017
	Rate	95%CI	Rate	95%CI	Rate	95%CI	Rate	95%CI	Rate	95%CI	Rate	95%CI	Rate	95%CI
< 1 years	3.6	0.5, 25.6	23.9	11.4, 50.1	25.3	12.1, 53.1	6	1.5, 23	4.5	0.6, 31.9	14	3.5, 56	8.7	5.6, 13.5
1–4 years	15.5	9.3, 25.7	23.7	15.9, 35.4	12.1	6.7, 21.8	6.4	2.9, 14.2	10.3	5.2, 20.5	3.2	0.8, 12.8	11.6	9.1, 14.8
5–14 years	52.3	41.8, 65.3	94.1	80.9, 110.5	54.6	44.3, 67.3	15.4	10.2, 23.2	47.3	35.9, 62.2	13.3	7.4, 24	49.5	44.9, 54.6
15–44 years	83.6	69.2, 101	109.5	94, 127.6	79.4	66.4, 94	26.9	20, 36.1	69.8	56.4, 86.4	59.5	45.3, 78.1	254.6	234.6, 276.3
> 45 years	14.4	6.9, 30.2	38.5	25.1, 59	51.9	36.1, 74.7	9.8	4.4, 21.8	31.3	18.9, 51.9	45.3	27.7, 73.9	41.8	34.1, 51.2
Total	59.8	52.2, 68.5	102.8	92.8, 113.9	68.3	60.4, 77.2	21.1	17, 26.2	42	35.9, 49	20.9	16.9, 25.9	324.1	305.9, 343.3

### Seasonality and threshold of medically attended influenza like illness (ILI)

**Fig. 5:**
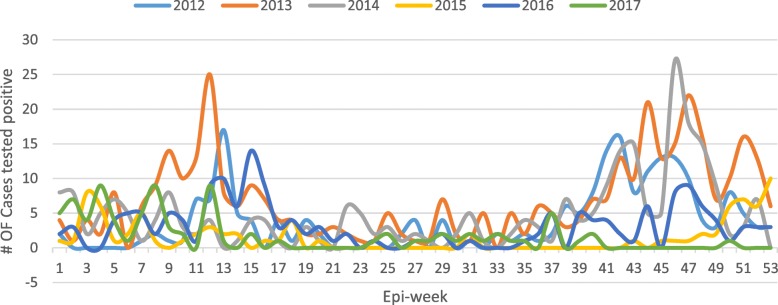
Trend of medically attended Influenza positive cases by year from 2012 to 2017, Ethiopia

**Fig. 6:**
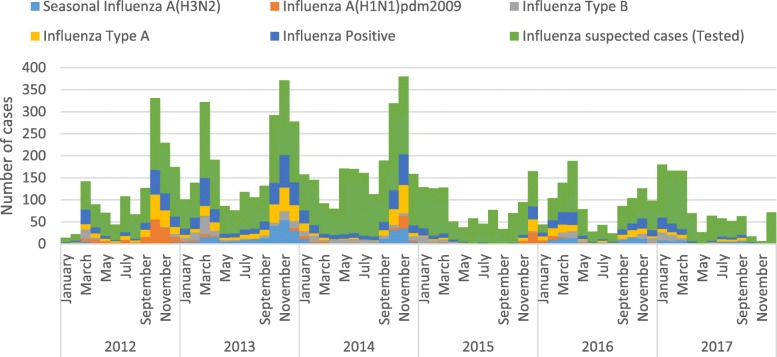
Monthly distribution of medically attended influenza virus-positive cases by subtype among Seasonal influenza patients in eight sentinel sites of Ethiopia, 2012–2017

The seasonality of medically attended influenza like illness had two peak seasons (Figs. 5 and 6, below). The highest number of influenza positive cases were in a period from October–December (week 39–51) and from April–June (week 7–15 of the year) in 8 years of surveillance period (2012–2017).

**Fig. 7:**
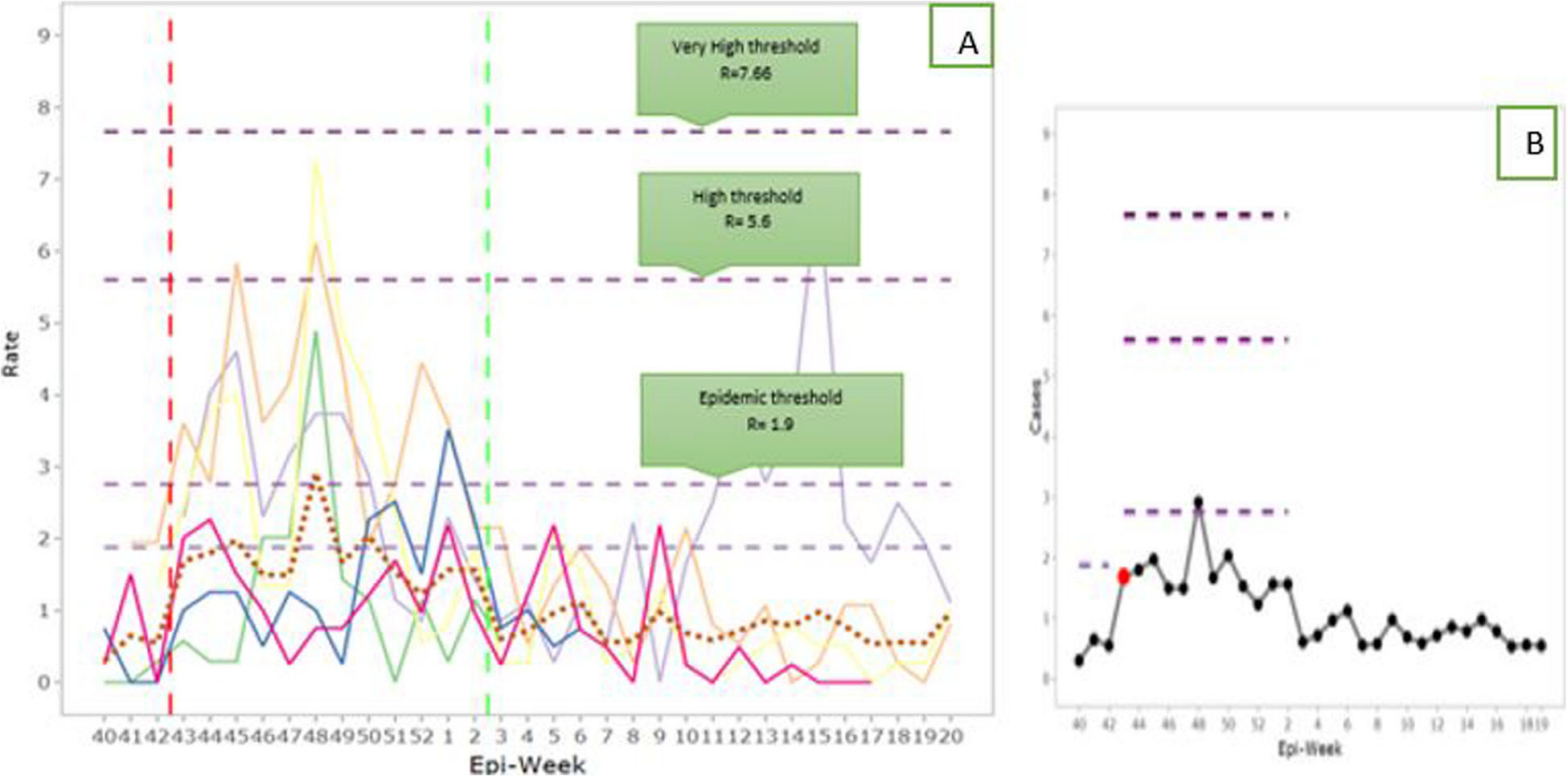
Seasonality and threshold (Very high, High and Epidemic) of medically attended influenza (ILI) in the lower administrative level catchment population, 2012–2017 (MEM epidemic curve and average curve)

**Fig. 8:**
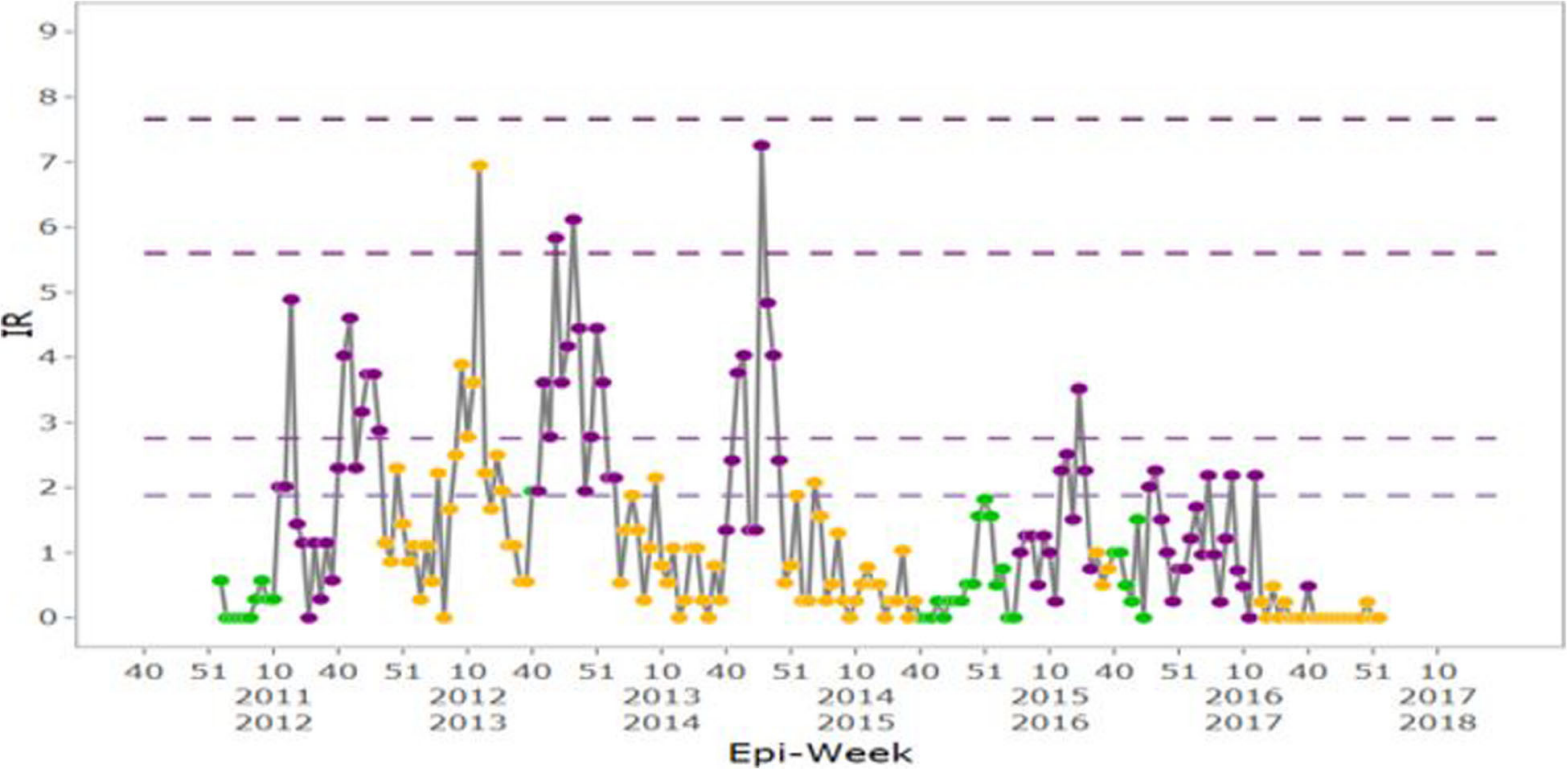
Time series of weekly detection of influenza like illness per year in Ethiopia and seasonal model (MEM application) from 2012 to 2017

The epidemic season mean start lies on week 42 (October) and the mean end lies week 2 (December) of the year. The highest threshold was recorded by the year 2016/17 (R = 8.2, r = 032) followed by 2014/15 (R = 7.9, r = 0.37) and 2012/13 (R = 7.5, r = 0.43). The average epidemic lengths might last for 11–12 weeks. The weekly epidemic threshold of influenza ranged in a Incidence rate of 2.5 (minimum threshold) to 7.66 (Very high threshold) per 100,000 population in the catchment area [Mathew correlation coefficient “r” of 3.4 with 0.42 sensitivity rate and 0.67 positive predictive value], Figs. 7 and 8, below.

## Discussion

Presenting country-level data from Ethiopia is incredibly useful, especially in a region where country-level data are not well described or published. The frequency distribution, influenza positive case proportion, incidence rate and seasonality of medically attended influenza (ILI and SARI) based on sentinel surveillance data (epidemiological and laboratory data) collected from eight sentinel sites over 6 years period (2012–2017) were presented. A total of 5715 medically attended influenza suspected patients who fulfills ILI and SARI case definition (77% ILI and 23% SARI) were enrolled in eight influenza sentinel surveillance sites located in four regions of Ethiopia from 2012 to 2017. The variation in the number of influenza suspected cases among sentinel sites and the case classification might be due to the difference in patient flow in the sites and effectiveness of the surveillance activities in each sentinel sites.

Over the 6 years period, the overall percent positive for medically attended influenza cases were 20% (1166/5715). The percent positive for ILI and SARI case were 25% (1130/4426) and 3% (36/1289), respectively. This finding is relatively higher than 16.2% posetivity rate in most of African countries and 18% positivity rate in eastern African countries as reported by WHO during 2016 [21]. Whereas, this shows higher compared to the influenza positivity rate from ILI samples in Niger (12%) and Gabon (11%), and lower compared to 23.3% posetivity rate in Madagascar [22–24]. The influenza positivity rate for SARI samples was 2.83% which was low compared to findings in South Africa and China which reported 8 and 6% respectively [25, 26]. In addition, the presented finding was lower in comparison with 16.5 and 20.2% influenza virus detection rate among SARI cases reported in Eastern Mediterranean Region and Vietnam [27, 28]. The variation if the findings might be due to the difference in accuracy of detection of cases at the sentinel sites, the timing of sampling, specimen quality and handling approach, effectiveness of the diagnostic testing in different settings. In contrast, it might be due to the true variation in circulating influenza viruses in different geographical areas as reported by different countries.

Off the total 1166 medically attended influenza positive cases 65% were influenza type A and 35% were influenza type B. Off the influenza type A cases the predominantly circulating influenza subtype 60% (*n* = 455) were Seasonal Influenza A(H3N2) and 39% (*n* = 293) were Influenza A(H1N1)pdm09. This finding is closely comparable with a finding reported by WHO in which, 56% of influenza A(H3N2) subtype and 32% A(H1N1) pdm subtype in most African countries [21]. Where us the present finding has different from 117 (38.0%) influenza A(H3N2) and 58 (19%) influenza A(H1N1)pdm09, reported in Madagascar [24].

In the present finding, the seasonality of influenza has two peak seasons in a period from October–December (week 39–51) and from April–June (week 7–15 of the year). Furthermore, the seasonality of influenza using cumulative Incidence rate with in the lower administrative level catchment population from 2012 to 2017 shows that the epidemic season mean start lies on week 42 (October) and the mean end lies week 2 (January) of the year. The highest threshold was recorded by the year2016/16 (R = 8.2, r = 032) followed by 2014/15 (R = 7.9, r = 0.37) and 2012/13 (R = 7.5, r = 0.43)). The average epidemic lengths might last for 11–12 weeks. Based on the applied model the weekly epidemic threshold of influenza ranges from 2.5 (minimum threshold) to 7.66 (Very high threshold) per 100,000 population in the catchment area [Mathew correlation coefficient “r” of 3.4 with 0.42 sensitivity rate and 0.67 positive predictive value]. This finding agreed with the WHO report in which the peak of transmission was observed from epi weeks 7 to 9 of 2016 and 2015, which coincided with the northern hemisphere winter and influenza type A was predominant. It also reported that there was a second increase in influenza activity from epi weeks 48 to 51 of 2016 predominated by influenza type B [21]. The present finding also aligns with the finding by Hirve et al. (2016) who reported a seasonality pattern similar to the northern hemisphere temperate regions start of the main influenza season between October and December [29].

The mean annual incidence rate of influenza (52.48 per 100,000) in the present finding is relatively higher in comparable to the finding reported in Kenya (2012–2014) with an estimated annual Incidence rate of hospitalized influenza-associated SARI was 21 per 100,000 population and the mean annual Incidence rate of non-hospitalized influenza-associated SARI was 81.7 (95% CI 74.1–89.9) per 100,000 persons [30]. Even if the present finding doesn’t entail on SARI, association of the non-hospitalized influenza associated SARI with the ILI indicates that the finding in the present finding is relatively lower. In addition, the present finding is higher than the estimated mean annual Incidence rate of 30.0 per 100,000 population reported in Madagascar [24].

The presented finding showed that the overall incidence rate of ILI were higher in the age group of 15–44 years of age [R = 254.6, 95% CI; 173.65, 335.55] followed by 5–14 years of age [R = 49.5, CI 95%; 31.47, 130.43]. The mean annual incidence rate of medically attended influenza cases showed variation in comparison to other countries report [24–30]. The variation in the reported incidence rate among the age group might be due to the actual difference in the incidence of influenza in different countries, the quality of surveillance system, the health care seeking behavior of the community and also it might be due to the different approach/method of analysis for calculating the incidence rate.

### Limitation of the study

The presented study has limitations that warrant discussion. The very low health care seeking behavior of the community for influenza infection might affect the findings of the estimated Incidence rate of influenza in Ethiopia. The adjustment factor for this low health seeking behavior and also to the national level were not applied in the present finding. The presented findings has only based on the surveillance data from eight sentinel sites and it does not provide has limitations in providing the burden at national level. The surveillance system has also enrolled only few SARI patients whereas the influenza positive case proportion of SARI as compared with total admission cases was high in all hospitals. The surveillance system is also capturing only influenza. Hence the estimates in this study should be considered minimum estimates.

## Conclusion

Influenza like illness has significant morbidity in Ethiopia, and that it is disproportionally distributed by age. The majority of medically attended influenza positive cases were influenza type A and higher number of it were seasonal influenza A(H3N2) and Influenza-A (H1N1) pdm2009. The incidence rate of influenza found to be higher in the age group of 5–14 years and 15- 44 years. Incidence rate of Seasonal influenza has two peak seasons in a period from October–December and from April–June. The rate of influenza is significant, warranting a response from government (public health agencies) to prevent, detect, and treat influenza in the name of national health security. It could be useful to policy makers to consider influenza seasonality when determining timing, vaccine composition, and target age groups for influenza vaccination campaigns. Improving the surveillance system could help further define populations at risk for influenza.

## Data Availability

The data sets and materials used to prepare this manuscript are purely routine surveillance data and available from the corresponding author anytime on reasonable request through: mussetad02@yahoo.com.

## Abbreviations

CDC: Center for Disease Control and Prevention
EPHI: Ethiopian Public Health Institute
HC: Health Center
ILI: Influenza like Illness
IQR: Interquartile range
IRR: Influenza Reagent Resource
MEM: Moving Epidemic Model
NIL: National Influenza Laboratory
PCR: Polymerase Chain Reaction
RNA: Ribonucleic acid
RT-PCR: Real Time Polymerase Chain Reaction
SARI: Severe Acute Respiratory Infection
VTM: Viral Transport Media
WHO: World Health Organization
